# The Chinese Translation Study of the Cognitive Reserve Index Questionnaire

**DOI:** 10.3389/fpsyg.2022.948740

**Published:** 2022-07-22

**Authors:** Ting Cao, Shifang Zhang, Mingming Yu, Xiaoyan Zhao, Qiaoqin Wan

**Affiliations:** Department of School of Nursing, Peking University, Beijing, China

**Keywords:** cognitive reserve, cognitive reserve index questionnaire, reliability, Chinese language, translation

## Abstract

**Purpose:**

The purpose of this study was to perform the translation and adaption of the Cognitive Reserve Index questionnaire into Chinese and assess the reliability of the Chinese version.

**Materials and Methods:**

The Chinese version of the Cognitive Reserve Index questionnaire was created from a standard forward-backward translation. A total of 371 volunteers, aged between 20 and 89 years, participated in this survey. Participants were divided into three age-groups (Young, Middle-aged, and Elderly), and subgroup differences were examined by independent samples *t*-tests, ANOVA analysis as well as *post-hoc* analysis. Pearson correlation analysis was applied to test the association between the total scores and each subscore (CRI-Education, CRI-WorkingActivity, and CRI-LeisureTime). The internal consistency and test-retest reliability of the Cognitive Reserve Index questionnaire were assessed. The test-retest reliability was measured among 40 participants with a 2-week interval using intraclass correlation coefficient.

**Results:**

Strong correlations were observed between the total scores and each subscore (CRI-Education, CRI-WorkingActivity, and CRI-LeisureTime: *r* = 0.65, 0.79, and 0.70, respectively). In contrast, it was found low to moderate correlations among three subscores. The internal consistency was acceptable (Cronbach's alpha coefficient = 0.68). The intraclass correlation coefficient for total scores of the Chinese version of the Cognitive Reserve Index questionnaire was 0.87 (95% CI 0.74–0.93).

**Conclusion:**

The Chinese version of the Cognitive Reserve Index questionnaire was a potentially reliable and practical tool for evaluating cognitive reserve accumulated through a person's life span.

## Introduction

Cognitive reserve is a hypothetical construct that has received growing attention in current studies (Stern, 2002). Unlike a static or passive model of brain reserve, cognitive reserve refers to the cumulative flexibility (i.e., adaptability, efficiency, and capacity) of cognitive processes generated by participating in a variety of mentally stimulating activities throughout a person's lifetime, which enables to explain differences in susceptibility of cognitive abilities or daily function to brain aging, pathology or insult (Stern et al., 2020). The cognitive reserve hypothesis holds that greater cognitive reserve can better withstand or even attenuate the effects of age-related changes and pathological damages on cognitive status through compensatory strategies and brain network, whereas a more rapid course of cognitive decline might be notable once the amount of healthy or functional neurons drops to a certain threshold (Stern et al., 2019). In 2013, Kivipelto et al. proposed the protection/risk model of cognitive impairment and suggested that protective factors (such as education level, physical exercise, cognitively stimulating activities, and social interaction), mainly through the mechanism of increasing cognitive reserve, could buffer the impact of risk factors (such as advanced age, hypertension, diabetes, and depression) on cognitive impairment (Kivipelto et al., 2018). Some studies have supported that high life span cognitive reserve was associated with better cognitive performance, and later onset of clinical manifestations in diseases, such as Alzheimer's disease (Xu et al., 2020), Parkinson's disease (Hindle et al., 2014), stroke (Umarova et al., 2019; Shin et al., 2020), traumatic brain injuries (Fraser et al., 2019), bipolar disorder (Hinrichs et al., 2017), and multiple sclerosis (Lopez-Soley et al., 2020).

Although many researchers accept the concept of cognitive reserve, the lack of precise quantified measures limits the epidemiological studies of cognitive reserve. A growing number of studies have attempted to estimate cognitive reserve by using single or various combinations of the following proxies, such as education, occupational attainment, engagement in cognitively demanding activities, and premorbid intelligence (Nucci et al., 2012; Cheng, 2016; Stern et al., 2019). Nucci et al. (2012) designed and standardized the Cognitive Reserve Index questionnaire (CRIq) to evaluate a person's cognitive reserve, which consists of three parts, namely, education, working activity, and participation in leisure activities. The CRIq assesses the frequency and the time spent in these cognitively demanding activities over the individual's lifetime. To date, the CRIq has been available in more than 15 different languages including Italian, English, French, German, Spanish, Portuguese, Greek, and Turkish at http://cri.psy.unipd.it, and it has also been used to assess cognitive reserve in diverse populations, such as healthy adults as well as patients with liver cirrhosis, Parkinson's disease, multiple sclerosis, subjective cognitive impairment, mild cognitive impairment, and Alzheimer's diseases (Kartschmit et al., 2019; Garba et al., 2020). However, the lack of a Chinese version of CRIq (C-CRIq) limits its potential use among Chinese populations. Therefore, this study aimed to translate CRIq into Chinese and test its reliability.

## Materials and Methods

### Instruments

The CRIq encompasses some demographic data (date and place of birth, gender, place of residence, marital status, etc.), and other three dimensions, namely, education (2 items), working activity (1 item), and leisure time (17 items), each of which calculate the frequency and the number of years of these items. Finally, the CRIq yields a total score, called the Cognitive Reserve Index (CRI), which is a measure of CR. The higher the CRI, the higher the CR. Additionally, each dimension returns a subscore, namely, CRI-Education, CRI-WorkingActivity, and CRI-LeisureTime. The CRI-Education section includes formal years of education as well as occupational courses or vocational training. The CRI-WorkingActivity section includes the level and total years of adulthood professions. Notably, five different levels of working activities are available, namely, low-skilled manual work, skilled manual work, skilled non-manual or technical work, professional occupation, and highly intellectual occupation. The CRI-LeisureTime section includes a variety of enrichment activities performed during spare time, such as reading newspapers or magazines, visiting a museum, and exercising. The instructions, administration, and automatic calculation form for CRIq are available at http://cri.psy.unipd.it. The total CRIq scores are divided into five grades, namely, low (<70), medium-low (70–84), medium (85–114), medium-high (115–130), and high (>130). In the original study, the inter-item correlation was reported as good (Cronbach's alpha coefficient = 0.73).

### Translation

After obtaining authorization for translation and use of CRIq from the original author, Dr. Massimo Nucci, a standard forward-backward translation approach was followed (Beaton et al., 2000). First, CRIq was independently translated from English into Mandarin Chinese by two authors (Cao and Zhang) of our study, and the discrepancies were discussed with a third researcher (Yu) until reaching a consensus. Second, this version was back-translated into English by two bilingual postgraduate students, who majored in translation and interpreting and were not familiar with the original questionnaire. Third, the professional researchers compared and verified that the C-CRIq was compatible with the original one in semantic and idiomatic terms. Finally, 30 volunteers were interviewed to administer the C-CRIq, and the results suggested that they had a fluent understanding of each item with no suggestions provided in the pre-final version stage. The C-CRIq is shown in Supplementary Material.

### Participants and Data Collection

A convenience sample of non-clinical volunteers was recruited from two cities (Beijing, Tianjin) in northern China and one Province (Jiangsu) in southern China from August 2021 to December 2021. Eligible subjects for the study comprised adults aged 18 years and older who could communicate in Chinese. Participants with a history of neurological or psychological disorder were excluded. The trained investigators (graduate nursing students) delivered the C-CRIq to volunteers face-to-face. The CRIq is not anonymous. However, if a participant refused to register his/her name, a fictitious name was generated. Of all the samples, 40 participants who agreed to register their names and telephone numbers were selected for a second round of testing C-CRIq 2 weeks later. After checking that there were no missing items, the completed questionnaires were retrieved on site. The survey process ensured informed consent, voluntary participation, and information confidentiality (Cebi and Kulce, 2021).

### Statistical Analysis

Data were expressed as mean with standard deviation (SD) for continuous or number and percentage for categorical data. Subgroup differences were tested with independent sample *t*-tests, One-way analysis of variance (ANOVA) as well as *post-hoc* analysis. A Pearson correlation analysis was applied to examine the relationship between the total and the subscores of C-CRIq. The Pearson's r correlation coefficients for all values were interpreted as follows: weak correlation (<0.3), moderate correlation (0.3–0.5), and strong correlation (>0.5; Cohen, 1988). The internal consistency reliability was assessed using Cronbach's alpha coefficient, and a value of ≥0.6 was considered to indicate adequate internal consistency (Pedroso and Gubert, 2021). The test-retest reliability was analyzed by intraclass correlation coefficient (ICC), which was calculated by the correlation between the first and second completion of C-CRIq. The test-retest reliability was stratified by ICC as follows: excellent (>0.80); good (0.60–0.80); moderate (0.40–0.60); and poor (<0.40) (Shrout and Fleiss, 1979). SPSS 24.0 was adopted for statistical analyses of all data. The statistical significance level was considered at *p* < 0.05.

### Ethical Approval

The study was approved by the Institutional Review Board (IRB) of Peking University (Approval No. IRB00001052-21125).

## Results

A total of 371 participants were included in our study (196 women, 52.8%). Their age ranged from 20 to 88 years (mean age: 52.3 ± 17.2, women: 47.3 ± 17.1, and men: 57.8 ± 15.5). All participants were classified into three age-groups according to the original work: Young, from 18 to 44 years (*n* = 140, 33.3 ± 5.6); Middle-aged, from 45 to 69 years (*n* = 160, 58.5 ± 7.1); and Elderly, from 70 to 88 years (*n* = 71, 75.6 ± 4.6). Their mean number of years of education was 12.5 ± 4.3 years (Young: 15.9 ± 3.4, Middle-aged: 11.2 ± 2.9, and Elderly: 8.9 ± 4.1; women: 13.3 ± 4.6 and men: 11.6 ± 3.9). ANOVA analyses revealed that the number of education years was significantly different between the age-groups (*F* = 114.607, *p* < 0.001), and *post-hoc* analysis showed that all binary group comparisons were significant (*p* < 0.01 for all). Women showed a higher level of education than men (*t* = −3.931, *p* < 0.001). The general demographics of the participants are presented in Table 1.

**Table 1:** Demographic characteristics of the participants.

	**Total**	**Young**	**Middle-Aged**	**Elderly**
Number of participants	371	140	160	71
Gender, *n* (female/male)	196/175	99/41	70/90	27/44
Age (years, M ± SD)	52.3 ± 17.2	33.3 ± 5.6	58.5 ± 7.1	75.6 ± 4.6
Education (years, M ± SD)	12.5 ± 4.3	15.9 ± 3.4	11.2 ± 2.9	8.9 ± 4.1
**Marital status**, ***n***
Single	44	40	4	0
Married	275	90	134	51
Divorced	27	10	14	3
Widowed	25	0	8	17

As for CRI-WorkingActivity, a total of 399 working activities were performed. Among 371 participants, 16 samples were unemployed or not yet employed, whereas 44 samples had two or three different levels of working activities throughout their life span. Low-skilled manual work was the most frequently reported activity (44.1%), followed by skilled manual work, skilled non-manual work, and professional occupation (22.7, 13.5, and 12.8%, respectively). Only 3.1% of the samples had a highly intellectual occupation. The frequencies of different levels of working activities across gender and age-groups are shown in Table 2. Regarding the leisure time activities, “grandchildren or elderly caring” (73.0%), “learning new technology” (67.1%), and “housework activities” (66.6%) were among the most frequently recorded activities. The least activities included participation in exhibitions, concerts or conferences, going to movies, and voluntary work (~6%).

**Table 2:** Frequencies of levels of working activity categories according to gender and age-groups.

**Working activity**	**Total (*n* = 415)**	**Gender**		**Age-groups**		
		**Females**	**Males**	**Young**	**Middle-Aged**	**Elderly**
		**(*n* = 216)**	**(*n* = 199)**	**(*n* = 153)**	**(*n* = 177)**	**(*n* = 85)**
Never employed	3.9%	6.0%	1.5%	9.2%	0.6%	1.2%
Low skilled manual work	44.1%	35.6%	53.3%	12.4%	62.1%	63.5%
Skilled manual work	22.7%	27.3%	17.6%	34.6%	15.8%	15.3%
Skilled non-manual work	13.5%	13.4%	13.6%	15.0%	13.6%	10.6%
Professional occupation	12.8%	14.4%	11.1%	24.2%	5.6%	7.1%
Highly intellectual occupation	3.1%	3.2%	3.0%	4.6%	2.3%	2.4%

The number and frequency distribution of CRI levels according to gender and age-groups are shown in Table 3. According to the distribution of CRI levels, 68.7% of participants (*n* = 255) were found to have a medium level of CRI, and 26.4% (*n* = 113) of participants had low-medium CRI. On the contrary, only 1.1% (*n* = 4), 2.7% (*n* = 10), and 1.1% (*n* = 4) of participants were found to have low, medium-high, and high CRI, respectively.

**Table 3:** The number and frequency distribution of CRI levels according to gender and age-groups.

**CRI levels**	**Total (*n* = 371)**	**Gender**		**Age-Groups**		
		**Females**	**Males**	**Young**	**Middle-Aged**	**Elderly**
		**(*n* = 196)**	**(*n* = 175)**	**(*n* = 140)**	**(*n* = 160)**	**(*n* = 71)**
Low	4 (1.1%)	3 (1.5%)	1 (0.6%)	0	0	4 (5.6%)
Medium-Low	98 (26.4%)	36 (18.4%)	62 (35.4%)	8 (5.7%)	54 (33.8%)	36 (50.7%)
Medium	255 (68.7%)	151 (77.0%)	104 (59.4%)	128 (91.4%)	99 (61.9%)	28 (39.4%)
Medium-High	10 (2.7%)	4 (2.0%)	6 (3.4%)	3 (2.1%)	4 (2.5%)	3 (4.2%)
High	4 (1.1%)	2 (1.0%)	2 (1.1%)	1 (0.7%)	3 (1.9%)	0

*CRI, cognitive reserve index*.

**Figure 1:**
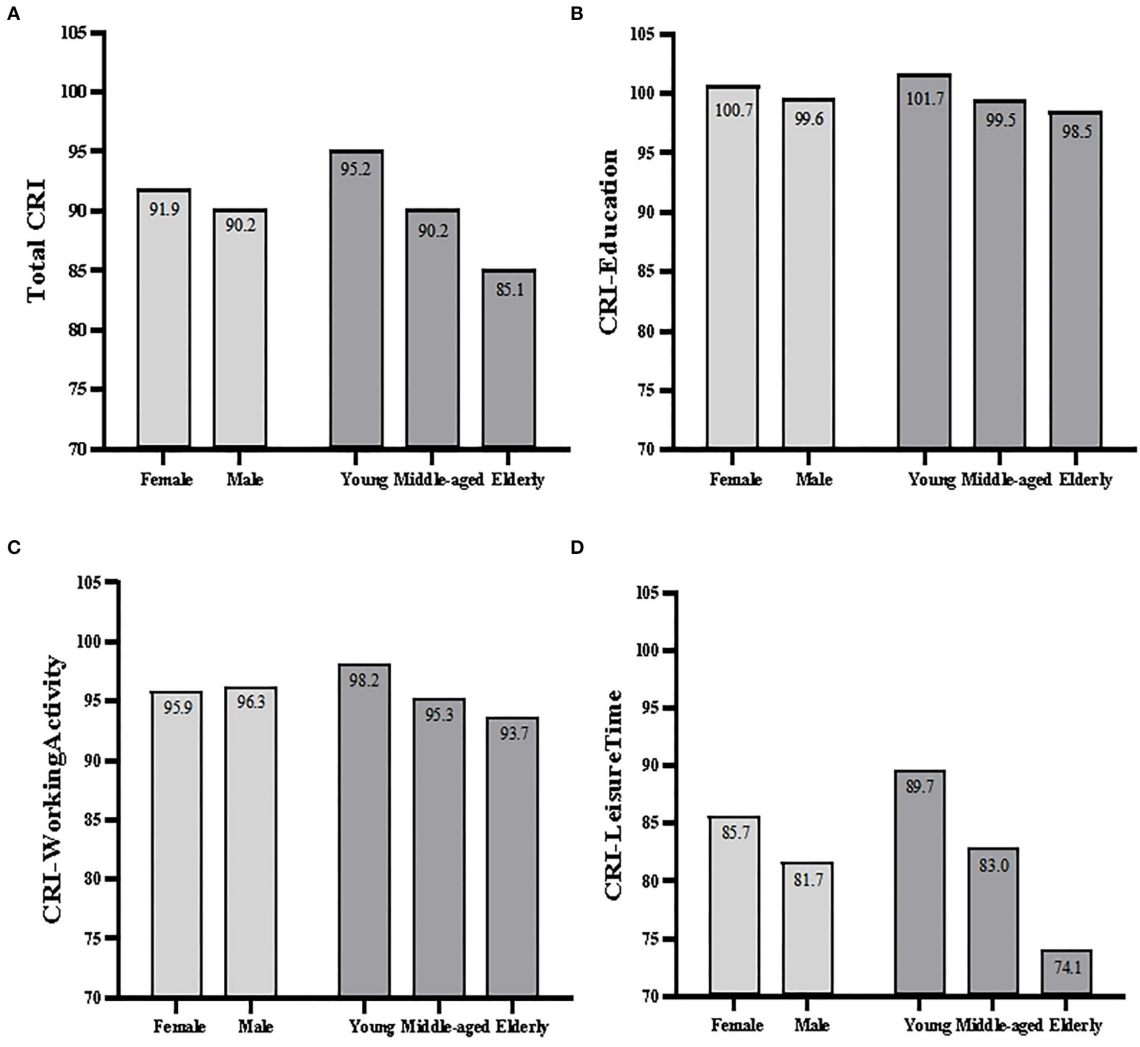
Bar plots of total CRI scores **(A)** and subscores across gender and age-groups **(B–D)**.

The mean total CRI scores and the subscores across gender and age-groups are presented in Table 4 and Figure 1. For total CRI scores, age was the only significant factor [Young: 95.2 ± 7.0, Middle-aged: 90.2 ± 11.6, Elderly: 85.1 ± 12.0, *F*_(2, 368)_ = 26.649, *p* < 0.001]. A *post-hoc* analysis indicated a significant difference in total CRI scores across the three age-groups (*p* < 0.01 for both). For the CRI-Education subscores, no significant difference was found in gender (*t* = −1.001, *p* = 0.317) and age-groups [*F*_(2, 368)_ = 2.367, *p* = 0.097]. For CRI-WorkingActivity subscores, age was the only significant factor [Young: 98.2 ± 6.5, Middle-aged: 95.3 ± 12.5, Elderly: 93.7 ± 15.9; *F*_(2, 368)_ = 5.240; *p* = 0.006]. The *post-hoc* analyses showed Young had higher CRI-WorkingActivity scores compared to the Middle-aged group (*p* = 0.031), whereas Young and Elderly groups as well as Middle-aged and Elderly groups did not reach significance. For CRI-LeisureTime subscores, women exceeded men (women: 85.7 ± 9.0, men: 81.7 ± 12.8, *t* = −3.462, *p* = 0.001) and the three age-groups were also significant [Young: 89.7 ± 6.6, Middle-aged: 83.0 ± 11.6, Elderly: 74.1 ± 9.6; *F*_(2, 368)_ = 81.958; *p* < 0.001]. A *post-hoc* analysis also showed a significant difference in CRI-LeisureTime scores across the three age-groups, with significantly higher scores in the Young group and the Middle-aged group than in the Elderly group (*p* < 0.001 for both).

**Table 4:** The mean and standard deviation of CRI scores for gender and age-groups.

	**Total (*n* = 371)**	**Gender**				**Age**				
		**Female**	**Male**	***t***	***P***	**Young**	**Middle-Aged**	**Elderly**	***F***	***P***
		**(*n* = 196)**	**(*n* = 175)**			**(*n* = 140)**	**(*n* = 160)**	**(*n* = 71)**		
Total CRI	91.1 ± 10.8	91.9 ± 10.0	90.2 ± 11.7	−1.543	0.124	95.2 ± 7.0	90.2 ± 11.6	85.1 ± 12.0	26.649	<0.001
CRI-Education	100.2 ± 10.4	100.7 ± 10.0	99.6 ± 10.8	−1.001	0.317	101.7 ± 11.0	99.5 ± 8.7	98.5 ± 12.3	2.367	0.097
CRI-Working Activity	96.1 ± 11.6	95.9 ± 10.4	96.3 ± 12.8	0.360	0.719	98.2 ± 6.5	95.3 ± 12.5	93.7 ± 15.9	5.240	0.006
CRI-Leisure Time	83.8 ± 11.1	85.7 ± 9.0	81.7 ± 12.8	−3.462	0.001	89.7 ± 6.6	83.0 ± 11.6	74.1 ± 9.6	81.958	<0.001

*CRI, cognitive reserve index*.

The findings detected that the total CRI scores had a strong correlation with three subscores: *r* = 0.65, 0.79, and 0.70 for CRI-Education, CRI-WorkingActivity, and CRI-LeisureTime, respectively. In contrast, it was observed low-to-moderate correlations among three subscores: *r* = 0.43 for CRI-Education and CRI-WorkingActivity, *r* = 0.20 for CRI-Education and CRI-LeisureTime, and *r* = 0.33 for CRI-WorkingActivity and CRI-LeisureTime. The Cronbach's alpha coefficient of the C-CRIq was 0.68. ICC for total CRI scores was 0.87 (95% CI: 0.74–0.93, *n* = 40).

## Discussion

The aim of this study was to translate the CRIq into Chinese and test the reliability of C-CRIq. All the questionnaires, instructions, and calculation forms were properly translated to the Chinese language, according to the original study.

In the literature, the concept of cognitive reserve stemmed from observed discrepancies between brain pathology and clinical manifestation and was a relatively novel concept (Stern, 2012). Due to the uncertainty about the exact nature of reserve, cognitive reserve was considered a latent variable, which can be inferred through its indicators (Stern et al., 2019). Since CRIq considered multiple proxies and gave standardized scores taking individuals' age into account, it has been widely used as a tool to evaluate cognitive reserve capacity. The results of this study also demonstrated that C-CRIq could be used as a practical and efficient tool for measuring cognitive reserve in Chinese samples. Consistent with previous studies (Nucci et al., 2012; Maiovis et al., 2016; Ozakbas et al., 2021), the total CRI scores were strongly correlated with three subscores, indicating each proxy's significant contribution to the total CRI. In addition, our inter-subscores correlations were low to moderate, reflecting the distinct information of each proxy. Our results indicated that the Cronbach's alpha coefficient of C-CRIq was 0.68, slightly lower than that of the original study (Cronbach's alpha coefficient = 0.73) (Nucci et al., 2012), but the internal consistency was acceptable. Likewise, the test-retest reliability of the C-CRIq was slightly lower than a very recent Turkey study (ICC = 0.95) (Ozakbas et al., 2021), but it also achieved a good stability and consistency (ICC = 0.87). The potential reason might be that CRIq, especially the CRI-LeisureTime section, was required to record the frequency and years of past activities, whereas the average age of the participants in our study was older (52.3 ± 17.2) than that of the Turkish study (39.5 ± 14.0), which may have resulted in a slight offset.

Mean total CRI score and subscores were similar across genders, except that women had existed higher scores than men in CRI-LeisureTime, possibly due to the fact that several items (e.g., housework, grandchildren, or elderly care) pertained specifically to women (Nucci et al., 2012). Indeed, housework activities (66.6%) and grandchildren or elderly care (73.0%) were the most frequently reported leisure activities in our study. Age significantly affected total CRI scores and subscores of CRI-WorkingActivity and CRI-LeisureTime. As predicted, the elderly group had the lowest total CRI scores and the two subscores. These findings may be explained by the fact that the elderly people in Chinese relatively have fewer years of education, as revealed in our study, and, in turn, less complex occupational lives (63.5% were in low-skilled manual work). In addition, the presence of physical health problems might be a factor in reducing their social activities. Contrary to an original study (Nucci et al., 2012), it was the Young group, not the Middle-aged group, that had the highest total CRI scores and subscores of CRI-WorkingActivity and CRI-LeisureTime. This might be explained that the Young group in our study had more complex work than the Middle-aged group, and only 9.2% of participants in the Young group in this study were unemployed, which was much lower than 24% in the original study.

According to the protection/risk model of cognitive impairment, increased cognitive reserve, which was resulted from protective factors, could buffer cognitive decline (Kivipelto et al., 2018). Current evidence indicated that higher cognitive reserve was prone to reduce the risk of symptoms onset of mild cognitive impairment by about 50% (Soldan et al., 2020). However, it was of great concern that only 2.7% (*n* = 10) and 1.1% (*n* = 4) of participants were found to have medium-high and high cognitive reserves in our study. Considering the potential benefit to individuals of cognitive reserve, it is therefore crucial to quantify individuals' cognitive reserve through CRIq and to take targeted preventive measures in advance. Notably, while CRIq existed in more than 15 different language versions of CRIq, adaptations of the original questionnaire with full English coverage were only available in Turkey, Greece, and the United States (Kartschmit et al., 2019; Garba et al., 2020). Meanwhile, it remained unclear whether and how these proxies of CRIq were fundamental to cognitive reserve as a construct (Stern et al., 2019). Future research needs to further test the relationship between the construct and its indicators.

Our study had several limitations. One of the major limitations was the nature of the sample. Despite aiming to achieve sample diversity, most participants in this study were from big cities. Therefore, future studies should include samples with relatively heterogeneous cultural and socioeconomic backgrounds and a more equal distribution among gender and age-groups to make the samples better representative of the Chinese population. Furthermore, the relatively small sample size could be listed as another limitation of this study, although it was somewhat comparable to the sample size of previous studies. Therefore, further research should include more participants.

Taken together, C-CRIq was easy to implement and exhibited acceptable internal consistency and satisfactory test-retest reliability in the context of Chinese society. The C-CRIq was easy to apply to different age-groups and the automatic calculation of the scores was quite time-saving, and it is considered a practical tool to be used.

## Conclusion

This study provided the translation and adaptation processing of the first Chinese tool to measure cognitive reserve. The current findings indicated that the C-CRIq was a reliable and time-saving tool for evaluating cognitive reserve. Chinese researchers can use the C-CRIq to quantitatively measure cognitive reserve and explore targeted rehabilitation measures for those with low cognitive reserve as early as possible, so as to reduce the risk of cognitive decline.

## Data Availability Statement

The raw data supporting the conclusions of this article will be made available by the authors, without undue reservation.

## Ethics Statement

The studies involving human participants were reviewed and approved by the Institutional Review Board (IRB) of Peking University. The patients/participants provided their written informed consent to participate in this study.

## Author Contributions

TC and SZ: resources, investigation, methodology, formal analysis, and writing—original draft. MY: methodology and writing—review and editing. XZ: investigation, methodology, and writing—original draft. QW: project administration, methodology, and writing—review and editing. All authors contributed to the article and approved the submittedversion.

## Funding

This study was supported by the National Key R&D Program of China (Grant No. 2020YFC2008804).

## Conflict of Interest

The authors declare that the research was conducted in the absence of any commercial or financial relationships that could be construed as a potential conflict of interest.

## Publisher's Note

All claims expressed in this article are solely those of the authors and do not necessarily represent those of their affiliated organizations, or those of the publisher, the editors and the reviewers. Any product that may be evaluated in this article, or claim that may be made by its manufacturer, is not guaranteed or endorsed by the publisher.
