# Supplementary material for: The Chinese Translation Study of the Cognitive Reserve Index Questionnaire
Source: Front Psychol. 2022 Jul 22;13:948740. doi: 10.3389/fpsyg.2022.948740 (PMC9353001; doi:10.3389/fpsyg.2022.948740)
Supplement: Supplementary file 1 [file Table_1.DOCX]

**认知储备指数问卷**

**指导语：**当参与者因确诊或怀疑认知能力下降而无法接受问卷调查时，CRIq可由其家庭成员或照顾者填写。在问卷适当的方格中打勾。

姓名：......................................

出生日期：........../........../.......... 出生地：...............................................

居住地：..................................................... 国籍：.....................................................

婚姻状况：未婚🞎 已婚🞎 离异🞎 丧偶🞎

**认知储备指数-教育**

**指导语：**每1年的教育计数为1。每6个月的职业培训课程计数为0.5。

年数

| 1.教育年限（包括研究生学习和任何专业） | ........ |
| --- | --- |
| 2.职业培训（每6个月计0.5） | ........ |

**认知储备指数-工作活动**

**指导语：**以5年为单位表示工作年限（0-5-10-15-20，等；例如，如果一人已经工作了17年，那么记录20）。工作活动按智力投入和个人责任感的程度划分为5个级别。如果同时从事多个工作，则记录所有的工作活动。

年数

| 1.低技能体力工作（农活、园丁、女佣、护理员、服务员、司机、修理工、管道工人、呼叫中心接线员，幼儿保姆等） | ........ |
| --- | --- |
| 2.有技能的的体力工作（工匠、厨师、商店店员、裁缝、推销员、军人（低级）、理发师、办公室职员、护士等） | ........ |
| 3.有技能的非体力工作（业务负责人、白领员工、销售代理、牧师或僧侣/尼姑、房地产中介、幼儿园教师、音乐家等） | ........ |
| 4.专业性职业（小公司总经理、律师、合格的自由职业者、承包商、医生、教师、工程师等） | ........ |
| 5.责任重大或高智商的职业（大公司的总经理、高级经理、法官、大学教授、外科医生、政治家等） | ........ |

**认知储备指数-休闲时间**

**指导语：**

·每个条目是指在整个成年生活中（即从18岁开始）规律进行的活动。

·本部分不包括所有有偿活动（对于有偿活动，返回认知储备指数-工作活动部分）。

·根据每项活动提到的频率（例如，每周、每月、每年）填写答案。

·“年数”一栏是指“经常/总是”进行上述活动的年数，以5年为尺度（5-10-15-20，等）进行扩大。例如，一个人经常阅读报纸27年，即使他/她已经停止阅读多年，也记录为“经常/总是”30年。

·如果该活动从不或很少进行（选择从不/很少），则无需注明年数。

·如果在参与者生命周期内，活动频率发生显著变化，则只记录最高频率的周期（以年为单位）。例如，如果一个人连续40年每天都开车，但是在接下来的15年里，他/她每周只开一两次车，那么答案“经常/总是”为40年。

**1.每周频率的活动**

≤2次/周 ≥3次/周 年数

| 1.阅读报纸和杂志 | 🞎从不/很少 | 🞎经常/总是 ........ |
| --- | --- | --- |
| 2.家务（做饭、洗衣、买食品杂货、熨衣服等） | 🞎从不/很少 | 🞎经常/总是 ........ |
| 3.开车（非骑自行车） | 🞎从不/很少 | 🞎经常/总是 ........ |
| 4.休闲活动（运动、打猎、跳舞、下棋、收集钱币等） | 🞎从不/很少 | 🞎经常/总是 ........ |
| 5.使用新技术（数码相机、电脑、互联网等） | 🞎从不/很少 | 🞎经常/总是 ........ |

**2.每月频率的活动**

≤2次/月 ≥3次/月 年数

| 1.社会活动（政党、休闲俱乐部、协会等） | 🞎从不/很少 | 🞎经常/总是 ........ |
| --- | --- | --- |
| 2.去电影院、剧院 | 🞎从不/很少 | 🞎经常/总是 ........ |
| 3.园艺、做手工、小规模操作如编织等 | 🞎从不/很少 | 🞎经常/总是 ........ |
| 4.照顾孙辈/侄女/侄子或年迈的父母 | 🞎从不/很少 | 🞎经常/总是 ........ |
| 5.志愿工作 | 🞎从不/很少 | 🞎经常/总是 ........ |
| 6.艺术活动（听音乐、唱歌、表演、绘画、写作等） | 🞎从不/很少 | 🞎经常/是 ........ |

**3.每年频率的活动**

≤2次/年 ≥3次/年 年数

| 1.参加展览、音乐会、会议 | 🞎从不/很少 | 🞎经常/总是 ........ |
| --- | --- | --- |
| 2.持续数天的旅行 | 🞎从不/很少 | 🞎经常/总是 ........ |
| 3.读书 | 🞎从不/很少 | 🞎经常/总是 ........ |

**4.固定频率的活动**

| 1.有孩子 | 🞎否 | 🞎是 数量........ |
| --- | --- | --- |

年数

| 2.照顾宠物 | 🞎从不/很少 | 🞎经常/总是 ........ |
| --- | --- | --- |
| 3.管理自己的活期帐户 | 🞎从不/很少 | 🞎经常/总是 ........ |

问卷被分发给：被调查者本人🞎 家人/照顾者🞎 .................................调查日期：........../........../.......... 调查者：........................................

**结果（得分）：**

认知储备指数-教育： ...................

认知储备指数-工作活动：...................

认知储备指数-休闲时间：...................

认知储备指数总分：...................

| 🞎 | 🞎 | 🞎 | 🞎 | 🞎 |
| --- | --- | --- | --- | --- |
| 低  <70 | 中-低  70~84 | 中  85~114 | 中-高  115~130 | 高  >130 |
